# Evaluation of [Cys(ATTO 488)^8^]Dermorphin-NH_2_ as a novel tool for the study of μ-opioid peptide receptors

**DOI:** 10.1371/journal.pone.0250011

**Published:** 2021-04-23

**Authors:** Despina Giakomidi, Mark F. Bird, John McDonald, Erika Marzola, Remo Guerrini, Serena Chanoch, Nidhuna Sabu, Barbara Horley, Girolamo Calo, David G. Lambert

**Affiliations:** 1 Department of Cardiovascular Sciences (Anaesthesia, Critical Care and Pain Management), University of Leicester, Leicester Royal Infirmary, Leicester, United Kingdom; 2 Department of Chemical and Pharmaceutical Sciences and LTTA, University of Ferrara, Ferrara, Italy; 3 Department of Pharmaceutical and Pharmacological Sciences, University of Padova, Padova, Italy; Cleveland Clinic Lerner Research Institute, UNITED STATES

## Abstract

The μ-opioid peptide (MOP) receptor is a member of the opioid receptor family and an important clinical target for analgesia. Measuring MOP receptor location and tracking its turnover traditionally used radiolabels or antibodies with attendant problems of utility of radiolabels in whole cells and poor antibody selectivity. To address these issues we have synthesized and characterised a novel ATTO488 based fluorescent Dermorphin analogue; [Cys(ATTO 488)^8^]Dermorphin-NH_2_ (Derm_ATTO488_). We initially assessed the binding profile of Derm_ATTO488_ in HEK cells expressing human MOP and CHO cells expressing human MOP, δ-opioid peptide (DOP), κ-opioid peptide (KOP) and Nociceptin/Orphanin FQ peptide (NOP) receptors using radioligand binding. Functional activity of the conjugated peptide was assessed by measuring (i) the ability of the ligand to engage G-protein by measuring the ability to stimulate GTPγ[^35^S] binding and (ii) the ability to stimulate phosphorylation of ERK1/2. Receptor location was visualised using confocal scanning laser microscopy. Dermorphin and Derm_ATTO488_ bound to HEK_MOP_ (pK_i_: 8.29 and 7.00; p<0.05), CHO_MOP_ (pK_i_: 9.26 and 8.12; p<0.05) and CHO_DOP_ (pK_i_: 7.03 and 7.16; p>0.05). Both ligands were inactive at KOP and NOP. Dermorphin and Derm_ATTO488_ stimulated the binding of GTPγ[^35^S] with similar pEC_50_ (7.84 and 7.62; p>0.05) and E_max_ (1.52 and 1.34fold p>0.05) values. Moreover, Dermorphin and Derm_ATTO488_ produced a monophasic stimulation of ERK1/2 phosphorylation peaking at 5mins (6.98 and 7.64-fold; p>0.05). Finally, in confocal microscopy Derm_ATTO488_ bound to recombinant MOP receptors on CHO and HEK cells in a concentration dependent manner that could be blocked by pre-incubation with unlabelled Dermorphin or Naloxone. Collectively, addition to ATTO488 to Dermorphin produced a ligand not dissimilar to Dermorphin; with ~10fold selectivity over DOP. This new ligand Derm_ATTO488_ retained functional activity and could be used to visualise MOP receptor location.

## Introduction

Opioid receptors are members of the seven transmembrane-spanning G protein-coupled receptor (GPCR) superfamily. The μ (MOP), δ (DOP) and κ (KOP) receptors are classical or naloxone sensitive and the Nociceptin/Orphanin FQ (N/OFQ) receptor (NOP) is naloxone insensitive. Whilst all opioid receptors are capable of the production of analgesia, the main target in the clinic is the MOP receptor. MOP receptors couple to G_i_/G_o_ G-proteins to enhance an outward potassium conductance to hyperpolarize, close voltage-sensitive calcium channels and inhibit adenylyl cyclase leading to the reduction of cAMP formation. In neurones this ultimately leads to reduced firing and neurotransmitter release [1–5].

The MOP receptor is widely distributed throughout the central nervous system and in neural and non-neural peripheral tissues [6]. Current methods to detect MOP receptor expression have several shortcomings. Use of radiolabels to study opioid receptors in native cells or tissue, where receptor densities are low is difficult due to the generally inadequate quantity of the sample that can be collected along with a relatively low specific activity of available radiolabels. Commercially available opioid receptor antibodies show poor selectivity and detection of mRNA does not necessarily indicate a functional protein [7–9]. There are a number of studies looking at turnover of tagged receptors. Typically these use receptors tagged with HA and FLAG but ultimately they require fixation and incubation with anti-HA or anti-FLAG antibodies [10, 11].

Dermorphin is a MOP receptor agonist isolated from the skin of the Amazon frog *Phyllomedusa sauvagei* in the early 1980s [12, 13]. Dermorphin binds to MOP with high affinity and an order of magnitude selectivity over DOP [14]. This relatively short (seven amino acids) peptide is easy to manipulate so we have used it as an acceptor for the fluorescent ATTO dye (488nm) to produce [Cys(ATTO 488)^8^]Dermorphin-NH_2_ (Derm_ATTO488_). The use of ATTO dyes leads to extended visualisation when compared to the more commonly used ALEXA dyes and linkage to bioactive peptides provides an increase in sensitivity when compared to use of antibodies or radioligand binding, particularly in low expression systems. Derm_ATTO488_ will have potential uses for tracking MOP receptors and when used in conjunction with other probes, for example N/OFQ_ATTO594_ [15] to examine opioid receptor interaction(s).

In this study we determine the binding properties of Dermorphin and Dermorphin_ATTO488_ along with functional activity in GTPγ[^35^S] binding and ERK1/2 phosphorylation at recombinant human opioid receptors expressed in HEK and CHO cells. Importantly we use Derm_ATTO488_ to visualise MOP expression in live CHO and HEK cells using confocal microscopy.

## Materials and methods

### Materials

Dermorphin and Naloxone were purchased from Sigma-Aldrich Co. (Dorset, U.K.). Naltrindole and Dynorphin A were from Tocris (Abingdon, U.K.). Tritiated Diprenorphine ([^3^H]-DPN) and tritiated N/OFQ ([^3^H]-N/OFQ) was from Perkin Elmer. Tissue culture media and supplements were from Sigma-Aldrich and Gibco. All other materials and reagents were of the highest purity available.

### Synthesis of [Cys(ATTO 488)8]Dermorphin-NH2 (DermATTO488)

**Fig 1:**
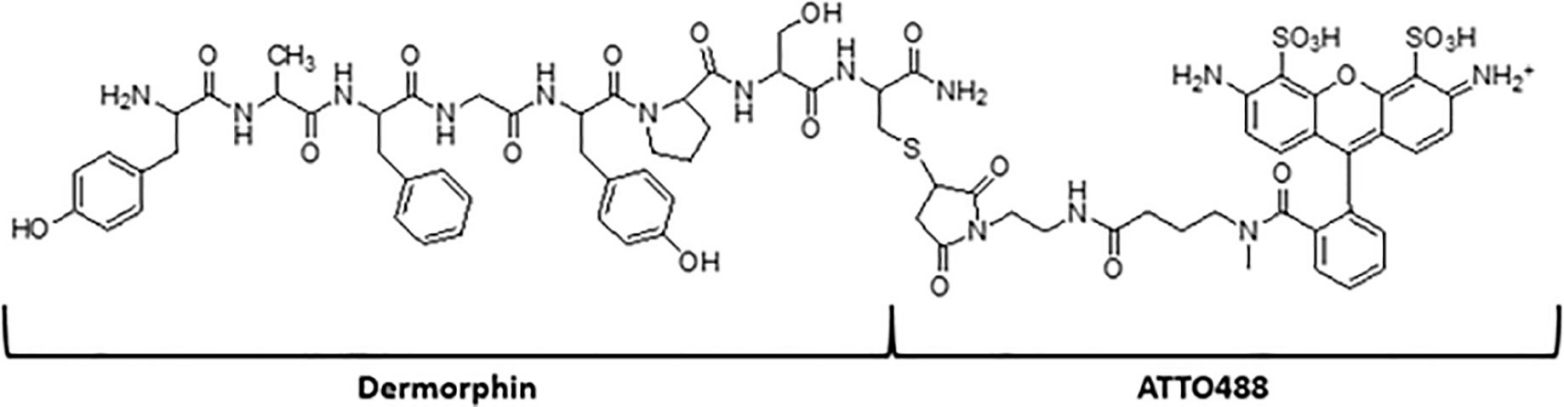
Chemical structure of Dermorphin-ATTO488.

The conjugation of [Cys^8^]Dermorphin-NH_2_ to ATTO 488 maleimide (purchased from ATTO-TEC GmbH Am Eichenhang 50 D-57076 Siegen Germany) was achieved using the classic thiol-Michael reaction. A solution of maleimide derivative fluorescent probe (1 mg, 1 equiv.) in CH_3_CN (250 μL) was added to a stirred solution of [Cys^8^]Dermorphin-NH_2_ (1.1 equiv.) in 250 μL of H_2_O, followed by the addition of 25 μL of NaHCO_3_ 5%. The mixture was stirred in the dark under a nitrogen atmosphere and at room temperature for 15 minutes. The reaction was monitored by analytical HPLC analysis and after completion, preparative HPLC purification of the reaction mixture. Quantitative yield is depicted in the supplement. S1 Fig in S1 File is an HPLC chromatogram and S2 Fig in S1 File is an MS spectra of the final product. Final product structure is shown in Fig 1.

### Cell culture

Human Embryonic Kidney (HEK) or Chinese Hamster Ovary (CHO) cells expressing recombinant human opioid receptors were cultured in MEM or HAM F-12 media respectively until >90% confluence. HEK_hMOP_ cells were prepared in house (plasmid coding for the MOP receptor was from https://www.cdna.org/files/data/OPR0M10000.pdf). CHO_hMOP_ and CHO_hKOP_ cells were provided by T Costa (Instituto Superiore di Sanita, Rome, Italy). CHO_hDOP_ were supplied by E Varga (University of Arizona, USA). CHO_hNOP_ were from GSK (Stevenage, Herts, UK). Media was supplemented with 10% foetal bovine serum, 100μg/ml penicillin, 100μg/ml streptomycin and 2.5μg/ml fungizone. Selection media used for stock cells expressing human recombinant classical opioid receptors (HEK_hMOP_, CHO_hMOP_, CHO_hKOP_ and CHO_hDOP_) was supplemented with 200μg/ml geneticin (G418). The selection media for cells expressing human recombinant NOP (CHO_hNOP_) also contained 200μg/ml hygromycin B. For the live-cell imaging experiments, cells were plated on ethanol-sterilised 25mm coverslips for 48h before use.

### Membrane protein preparation

HEK_hMOP_, CHOP_hMOP_, CHO_hNOP_, CHO_hKOP_ and CHO_hDOP_ cells were harvested in harvest buffer consisting of 154mM NaCl, 10mM HEPES and 1.7mM EDTA, pH 7.4. Cells were homogenised (Ultra-Turrax T25 cell homogeniser) and membrane fragments were collected by centrifugation at 13,500rpm for 10min at 4 ^0^C. Membranes were resupended in fresh harvest buffer and the process was repeated once more. Finally, membranes were suspended in the required volume of assay buffer (0.5% BSA in 50mM Tris, pH 7.4). Protein concentration was determined using the Lowry method. For the GTPγ[^35^S] binding assay, after harvesting the cells were suspended in homogenisation buffer consisting of 50mM Tris-HCl and 0.2mM EGTA, pH 7.4 and at the final step, membrane fragments were suspended in assay buffer consisting 50mM Tris, 0.2mM EGTA, 100mM NaCl and 1mM MgCl_2_, pH 7.4.

### Radioligand displacement assays

40μg of freshly prepared membrane protein was suspended in 0.5ml of buffer containing 50mM Tris, 0.5% BSA and ~0.8nM [^3^H]-Diprenorphine (DPN) (for HEK_hMOP_, CHO_hMOP_, CHO_hKOP_ and CHO_hDOP_) or ~0.8nM [^3^H]-N/OFQ (for CHO_hNOP_) and varying concentrations (1pM-1μM) of unlabelled Dermorphin or Derm_ATTO488._ Non-specific binding was evaluated with 10μM naloxone for cells expressing the classical opioid receptors and 1μM N/OFQ for CHO_hNOP_. Reference ligands were added as indicated in the results. After 1h incubation at room temperature reactions were terminated by vacuum filtration onto a PEI-soaked Whatman GF/B filter paper using a Brandel harvester [14, 16].

### GTPγ[^35^S] binding assay

40μg of freshly prepared membrane protein was suspended in 0.5ml of 50mM Tris-HCl buffer containing 0.2mM EGTA, 100mM NaCl, 1mM MgCl_2_, 0.1% BSA, 0.15mM Bacitracin, 33mM GDP, ~240pM GTPγ[^35^S] and varying concentrations (1pM-1μM) of unlabelled Dermorphin or Derm_ATTO488_. Non-specific binding was determined with 10μM unlabelled GTPγS. Membranes were incubated for 1h at 30°C with gentle agitation. Reactions were terminated by vacuum filtration onto dry Whatman GF/B filter paper using a Brandel harvester [14, 16].

### Western blotting–detection of ERK1/2 phosphorylation in HEK cells

HEK_hMOP_ cells were grown in 6-well plates coated with poly D lysine (0.1mg/ml) and serum starved for 24h. Cells were washed with Krebs buffer preheated to 37°C (118mM NaCl, 4.7mM KCl, 1.2mM KH_2_PO_4_, 1.2mM MgSO_4_, 11.9mM Glucose, 10mM HEPES, 1.3mM CaCl_2_, pH 7.4) for 30min. Dermorphin and Derm_ATTO488_ are subsequently added at a final concentration of 1μM and incubated for varying times (1min, 2.5min, 5min, 7.5min, 10min, 15min and 30min). Time zero was included as a control (no drug). Incubation was terminated by rinsing with ice cold PBS and the addition of lysis buffer (20mM Tris-HCl, 137mM NaCl, 2mM EDTA, glycerol, 10% Triton X-100, 0.054g b-Glycerophosphate, 0.018g sodium orthovanadate, 0.8μM pepstatin A, 500μM Leupeptin, 0.4mM benzamidine and 0.4mM PMSF). Reactions were centrifuged at 13,000rpm for 15min at 4 ^0^C then an equal volume of 2x sample buffer (Tris-HCl pH 6.8, 10% SDS, 38mM Glycine, 200mM DTT, bromophenol blue) was added and samples were heated to 95°C for 5min. 50μl of each sample, along with protein marker (biotinylated protein ladder #7727 Cell signalling technology) was loaded onto an 10% SDS-PAGE gel. Proteins were separated by electrophoresis at 150V for 1h in 1X Running buffer (Bio-Rad Laboratories). Proteins were transferred onto a nitrocellulose membrane (0.45μm, Thermo Fisher Scientific) in blotting buffer (39mM glycine, 48mM Tris-HCl, 0.037% SDS, 20% methanol) overnight. The membrane was stained with Ponceau S solution (1% w/v Ponceau in 1% Acetic Acid) for 3min on a rocker at room temperature. The membrane was then washed with dH_2_O and TBST buffer (5M NaCl, 1M Tris-HCl pH 7.5, 0.05% Tween-20) and soaked in TBST-10% milk for 2h on a shaker. Monoclonal phospho-p44/42 MAPK (ERK1/2) antibody (dilution 1:6000, #4377S, Cell Signalling Technology) or Vinculin antibody (dilution 1:1200, #4650S, Cell Signalling Technology) were added to the membrane and incubated overnight at 4°C on a shaker. The anti-vinculin antibody was used as a control to ensure equal loading across the wells and proper transfer to the nitrocellulose membrane. Membrane was washed x5 with TBST and polyclonal anti-rabbit IgG (#A6154, Sigma Aldrich) or anti-biotin HRP (#7075, Cell Signalling Technology) was added. After 1h of incubation the membrane was washed, treated with Amersham ECL western blotting detection kit (RPN 2109, GE Healthcare) for 1min and bands were visualised using a BioRad Chemi-Doc MP Imager [14].

### Live-cell imaging

Coverslip cultures of CHO or HEK cells were placed on a Harvard Peltier plate and perfused with Krebs buffer, pH 7.4 at 4°C (low temperature selected because Derm_ATTO488_ is an agonist). Derm_ATTO488_ was injected onto coverslips at four different cumulative concentrations; 1nM, 10nM, 100nM or 1μM. Cells were incubated with the lowest Derm_ATTO488_ concentration for 3mins, washed with ice-cold Krebs buffer for 2min then imaged. After a further wash of 1mins in HEK cells or 3min in CHO cells (to produce z-stack) the next concentration was added and imaged as described. To define non-specific label binding cells were incubated with 25μM of unlabelled Dermorphin for 5min and then with 1μM of Derm_ATTO488_ added as above. Additional experiments were also performed using 15μM Naloxone to determine NSB. Images were taken using an oil immersed 60x objective in a Nikon C1Si microscope. Both HEK and CHO cells were imaged using the 488nm wavelength laser with a 20% power setting and a 7.15 gain-green channel. The laser and gain settings were maintained constant in both cell lines. Images were collected by the Nikon C1Si software. For the CHO cells only, due to the low signal observed, images were captured using the ‘z-stack project’. All images were analysed using FIJI. Four random regions of interest (ROI) were selected and averaged for background fluorescence. For concentration fluorescence relationship analysis total corrected cell fluorescence was calculated according to the equation: Integrated density–(Area of selected cell X Mean fluorescence of background Readings) [15].

### Data analysis

Data are expressed as mean±SEM for (n) individual experiments. In confocal experiments representative images are depicted from 5 independent experiments. All curve fitting was performed using Graphpad Prism 7. In radioligand experiments, ligand affinity (pK_i_) was estimated from the concentration producing 50% displacement corrected for the competing concentration of label according to Cheng and Prusoff [17]. In GTPγ[^35^S] assays, data are presented as a stimulation factor, that is the fold change in GTPγ[^35^S] binding relative to the basal [14, 16]. The concentration of drug producing 50% of the maximum response (pEC_50_) and the maximum response (E_max_) are shown. Statistical analysis (as noted in text and Display legends) was via ANOVA with post-hoc correction and/or t-test as appropriate with P values <0.05 considered significant.

## Results

We initially assessed binding affinity and selectivity before confirming ATTO conjugation retained biological activity in GTPγ[^35^S] binding assay and ERK1/2 stimulation. Once this basic profile was confirmed live cell imaging was used to visualise cell surface binding.

### [^3^H]-DPN / [^3^H]N/OFQ displacement to determine affinity and selectivity

**Fig 2:**
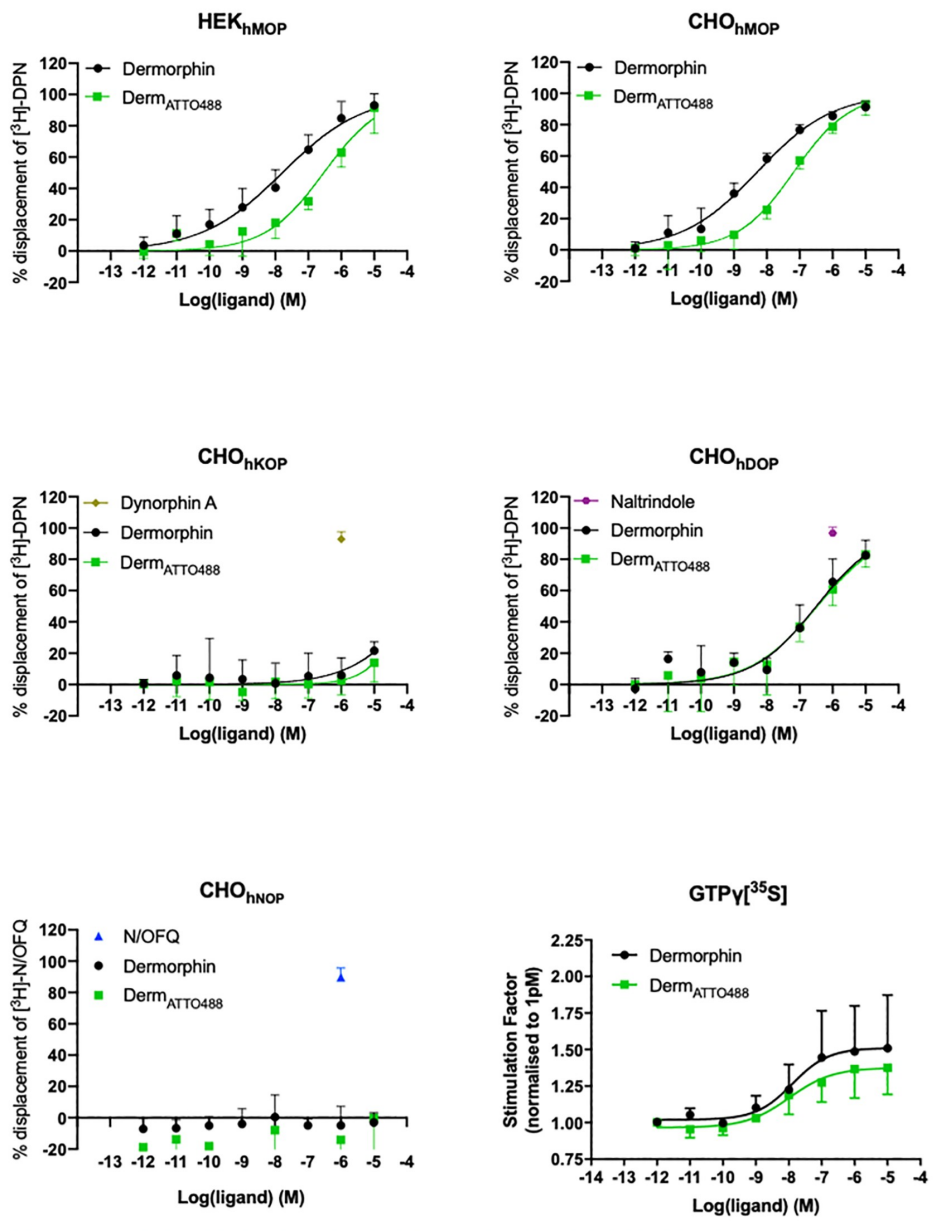
[^3^H]-DPN displacement assay and GTPγ[^35^S] activation assay. Displacement of [^3^H]-DPN by Dermorphin, Derm_ATTO488_, N/OFQ, Dynorphin A and Naltrindole in (A) HEK_hMOP_, (B) CHO_hMOP_, (C) CHO_hKOP_, (D) CHO_hDOP_ and (E) CHO_hNOP_. Data are presented as mean (SEM) of n = 10 experiments for the HEK cells and n = 6 for CHO cell lines. pK_i_ values are shown in Table 1. Ligand stimulated GTPγ[^35^S] binding by Dermorphin and Derm_ATTO488_ at CHO_hMOP_ (F). Data are presented as mean (±SEM) of n = 4 experiments. pEC_50_ and E_max_ values are shown in Table 2.

In HEK_hMOP_ and CHO_hMOP_, Dermorphin and Derm_ATTO488_ displaced the binding of [^3^H]-DPN in concentration dependent manner. In CHO_hMOP_ Derm_ATTO488_ displayed decreased binding affinity (pK_i_ 8.12) compared to Dermorphin (pK_i_ 9.26; p<0.05) Fig 2, Table 1. A reduction in affinity was also observed in HEK_hMOP_ (pK_i_ 7.00 and 8.29 for Derm_ATTO488_ and Dermorphin respectively; p<0.05) Fig 2, Table 1. In CHO_hDOP_ Dermorphin (pK_i_ 7.03) and Derm_ATTO488_ (pK_i_ 7.16) displaced the radioligand with similar affinity (p>0.05), Fig 2, Table 1. The pK_i_ values for Naltrindole, Dynorphin A and N/OFQ were previously published [14] so, in this study a single concentration (1μM) was used as a reference and this produced near full displacement. Both Dermorphin and Derm_ATTO488_ failed to displace [^3^H]-DPN in CHO_hNOP_ and CHO_hKOP_, and [^3^H]N/OFQ in CHO_hNOP_ cells Fig 2, Table 1. Based on CHO cell data Dermorphin and Derm_ATTO488_ showed 172 and 9 fold selectivity for MOP over DOP receptors and were inactive at KOP and NOP.

**Table 1:** Radioligand binding data.

pK_i_					
	HEK_hMOP_	CHO_hMOP_	CHO_hDOP_	CHO_hKOP_	CHO_hNOP_
**Dermorphin**	8.29±0.18	9.26±0.13	7.03±0.17	<5	<5
**Derm_ATTO488_**	7.00±0.10*	8.12±0.06*	7.16±0.23	<5	<5

Radioligand derived pK_i_ values for Dermorphin and Derm_ATTO488_ binding to MOP, DOP, KOP and NOP receptors as shown in Fig 2. Data are shown as mean±SEM of n = 10 for HEK_MOP_ and n = 6 for the CHO cells.

*p<0.05 (unpaired t-test) compared to Dermorphin.

**Table 2:** GTPγ[^35^S] binding in CHO_hMOP_ cell membranes.

	pEC_50_	E_max_
**Dermorphin**	7.84±0.23	1.52±0.36
**Derm_ATTO488_**	7.62±0.22	1.34±0.17

GTPγ[^35^S] activation in CHO_hMOP_ cells by Dermorphin and Derm_ATTO488_. Data show potency (pEC_50_) and efficacy (E_max_) derived from the data in Fig 2 and are presented as mean±SEM of n = 4 experiments. There were no statistically significant differences (p>0.05; unpaired t test).

### GTPγ[^35^S] binding assay

In CHO_hMOP_ Dermorphin and Derm_ATTO488_ stimulated GTPγ[^35^S] binding in a concentration dependent and saturable manner. The maximal response of Derm_ATTO488_ (E_max_ 1.34) was not significantly different (p>0.05) to that calculated for Dermorphin (E_max_ 1.52), Fig 2, Table 2. Derm_ATTO488_ and Dermorphin were equipotent (pEC_50_ 7.84 compared to 7.62; p>0.05), Fig 2, Table 2.

### Stimulation of ERK1/2 phosphorylation

**Fig 3:**
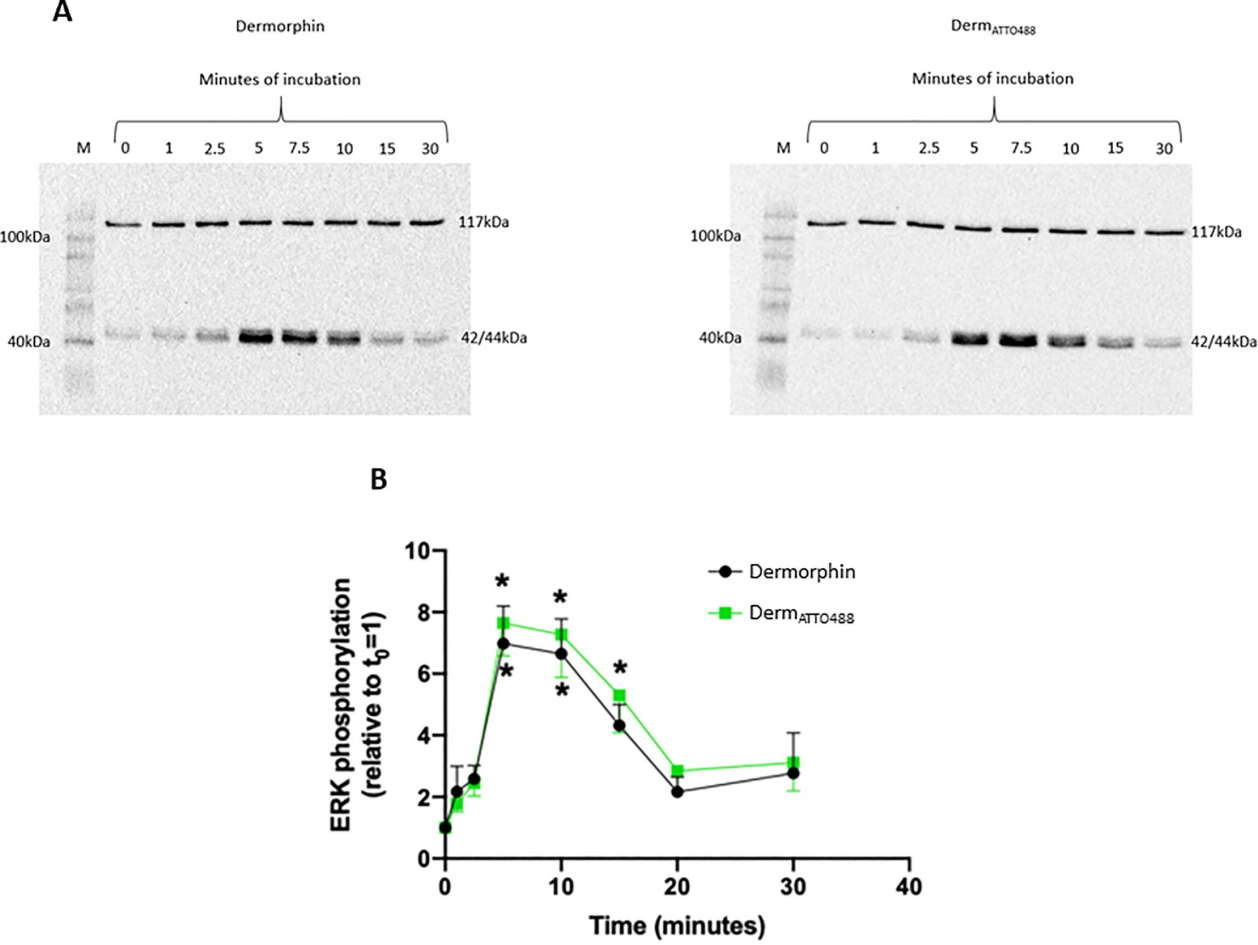
Activation of ERK1/2. (A) Representative blots for stimulation of phosphorylated ERK1/2 in HEK_hMOP_ by Dermorphin and Derm_ATTO488_. The 117kDa band corresponds to the vinculin protein used as a housekeeper and the ~42-44kDa band to phosphorylated ERK1/2 (the uncropped and unadjusted images are provided in S3 Fig in S1 File). (B). Time-dependent ERK1/2 phosphorylation by Dermorphin and Derm_ATTO488_. Data are presented as mean±SEM for n = 7. *p<0.05 compared to baseline according to ANOVA followed by Dunnett’s test for multiple comparison.

A time-response curve in HEK_MOP_ showed that stimulation by 1μM of Derm_ATTO488_ led to a monophasic pattern of ERK1/2 phosphorylation peaking at 5min and returning towards baseline by 20mins. The peak activity was statistically significant compared to t = 0 and was 7.64±1.06 fold greater. Similar data were obtained for Dermorphin (6.98±1.22 fold). These maximum stimulation values were not significantly different from each other (p>0.05). The phosphorylation pattern for Dermorphin and Derm_ATTO488_ were essentially superimposable, Fig 3 (S3 Fig in S1 File shows the uncropped and unadjusted images of the blots).

### Live-cell imaging

**Fig 4:**
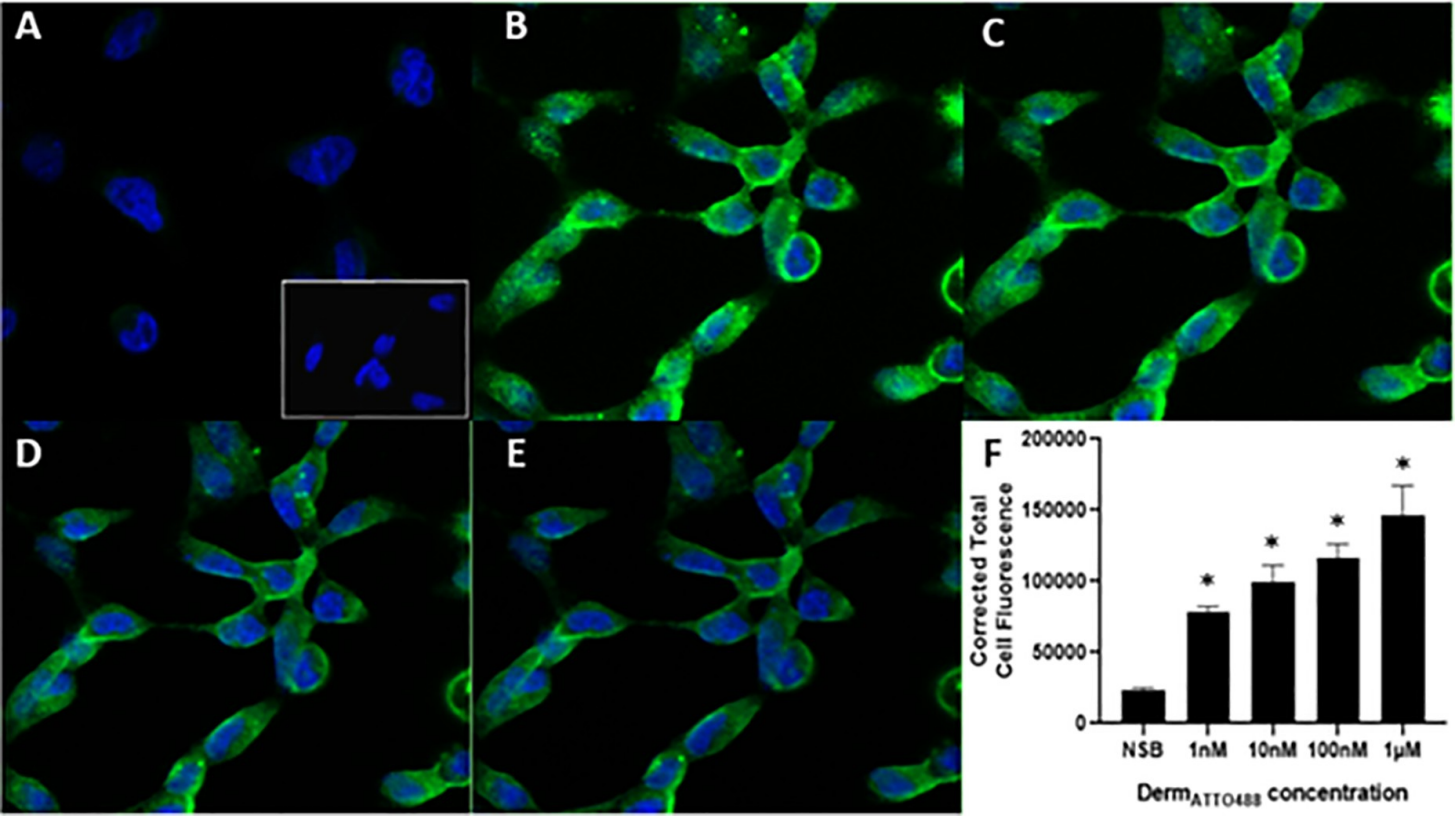
Concentration-dependent binding of Derm_ATTO488_ to MOP receptors expressed in HEK cells using confocal microscopy. Representative images of the binding of various concentrations (B: 1μM, C: 100nM, D: 10nM and E: 1nM). Nuclei are labelled (blue) with DAPI. Unlabelled (25μM) Dermorphin blocked the binding of 1μM Derm_ATTO488_ (16%) to the MOP receptors (A). Untransfected cells labelled 1μM Derm_ATTO488_ are depicted in A-inset. Image F shows mean corrected total cell fluorescence ± SEM for n = 5 experiments and *p<0.05 compared to NSB according to ANOVA followed by Dunnett’s test for multiple comparison.

**Fig 5:**
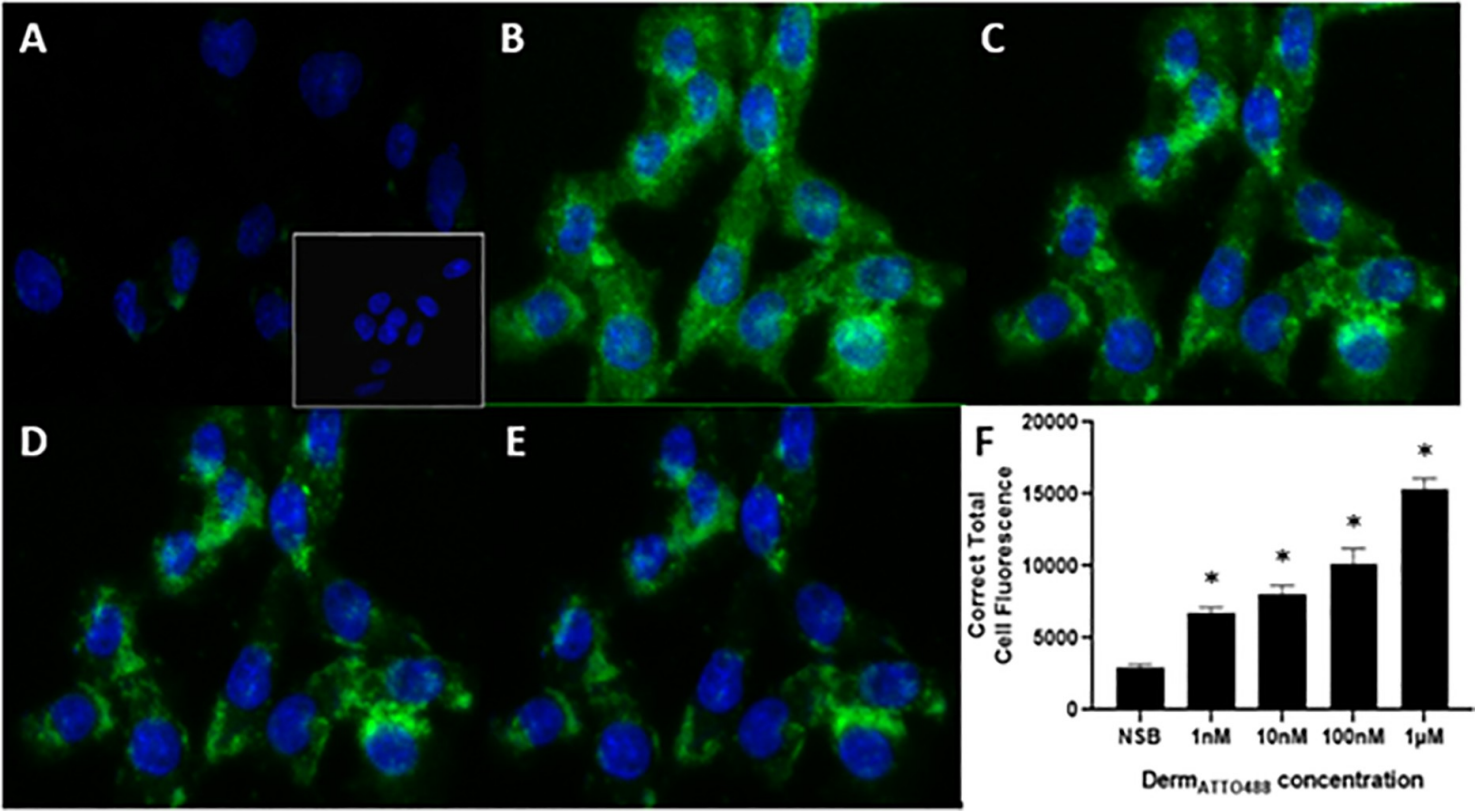
Concentration-dependent binding of Derm_ATTO488_ to the MOP receptors expressed in CHO cells using confocal microscopy. Representative images of the binding of various concentrations (B: 1μM, C: 100nM, D: 10nM and E: 1nM). Nuclei are labelled (blue) with DAPI. Unlabelled Dermorphin (25μM) blocked the binding of 1μM Derm_ATTO488_ (19%) to the MOP receptors (A). Untransfected cells labelled 1μM Derm_ATTO488_ are depicted in A-inset. Image F shows mean corrected total cell fluorescence ± SEM for n = 5 experiments and *p<0.05 compared to NSB according to ANOVA followed by Dunnett’s test for multiple comparison.

Four different concentrations of Derm_ATTO488_ (1nM, 10nM, 100nM and 1μM) were added to live HEK_MOP_ and CHO_hMOP_ cells and binding was measured using confocal microscopy and a 488nm laser, Figs 4 and 5. In all images nuclei are labelled with DAPI (blue) and the binding of Derm_ATTO488_ is shown in green. In both cell lines the binding was concentration dependent with the highest cell surface binding measured at the highest concentration used (1μM). Typical images are shown with average corrected total fluorescence for n = 5 independent experiments. Specificity of Derm_ATTO488_ (1μM) binding in single transfected lines (note; only MOP) was assessed by 5 min pre-incubation with an excess of unlabelled Dermorphin (25μM). As can be seen in Figs 4A and 5A there was demonstrable non-specific binding and this amounted to 16% and 19% in HEK_hMOP_ and CHO_hMOP_ respectively. Pre-incubation with unlabelled Naloxone (15μM) also defined non-specific binding in both HEK_hMOP_ (16%) and CHO_hMOP_ (20%) (S4 and S5 Figs in S1 File). In untransfected wild type HEK and CHO cells Derm_ATTO488_ (1μM) bound at ultra-low levels, comparable to that defined by unlabelled Dermorphin and Naloxone (Figs 4A-inset and 5A-inset).

## Discussion

In this study we have shown that conjugation of the MOP receptor agonist Dermorphin with the ATTO488 fluorophore produced a peptide ligand with slightly reduced binding affinity. Derm_ATTO488_ retained functional activity in GTPγ[^35^S] binding and ERK1/2 phosphorylation with effects superimposable to those of the native peptide Dermorphin. The new ligand has ~10fold selectivity over DOP and is inactive at KOP and NOP opioid receptors. This new ligand could be effectively used to visualise MOP receptors in live cells. This is a significant advantage over the use of radiolabels with their attendant problems, the generally poor selectivity of commercially available antibodies and the need to employ tagged receptors.

Physical-chemical characterization of ATTO488 is extensive and resulted in fluorophore characteristics for use in localization-based super-resolution imaging [18]. Moreover, ATTO dyes are particularly useful as they exhibit prolonged fluorescence and reduced photobleaching properties [19]. For a synthetic perspective, the fast and high yield reactivity of the ATTO488 maleimide derivative in very mild conditions (room temperature, acetonitrile/water as a solvent) with thiol groups (like the–SH of Cys side chain) make this dye an excellent candidate for easy conjugation with peptide sequences.

The binding affinity of Dermorphin at MOP was 8.29 (HEK) and 9.26 (CHO) representing just under a 10-fold difference. The difference between CHO and HEK (higher affinity in HEK) is difficult to explain but could result from a larger proportion of receptors in the G-protein coupled state as this has higher affinity for agonists [20]. In a previous paper [14] using Dermorphin in CHO cells we reported pK_i_ values at MOP of 8.69 (4-fold weaker than the current data); pK_i_ at DOP was essentially identical and there was no activity at KOP or NOP. In this paper we reported GTPγ[^35^S] binding and ERK1/2 phosphorylation [14]. A further potential explanation for the discrepancy in binding affinities could be differential coupling of MOP to additional protein(s) in CHO and HEK cells. Opioid receptors are known undergo extensive protein-protein interactions [21]. One such protein that has gained interest with respect to opioid receptor trafficking is Filamin A, a protein that couples membrane proteins to actin. However with respect to our data, Onoprishvili et al [22] showed that the binding of [^3^H]Diprenorphine and [^3^H]DAMGO were identical in M2 melanoma cells that do not express Filamin A and its subclone A7 transfected with this protein.

A key factor in using any receptor probe (fluorescent or radiolabelled) is to determine specificity of binding; or more specifically to define low non-specific binding (NSB). In these experiments we pre-incubated live cultures with the fluorophore parent; Dermorphin (an agonist) or the non-selective opioid receptor antagonist naloxone. In both cases Derm_ATTO488_ binding was displaced such that at 1μM labelling concentrations only 16–20% of it was non-displaceable. NSB was at the higher end in CHO compared to HEK cellular background. To add a further layer of definition, untransfected HEK and CHO cells were incubated with 1μM Derm_ATTO488_; in the absence of MOP receptors, probe binding was low and comparable (raw fluorescence) to that observed in the traditional displacement based NSB definition.

We have also reported previously on the conjugation of ATTO594 (red) to the NOP ligand N/OFQ. Unlike conjugation of ATTO488 to Dermorphin this conjugation produced a fluorescent ligand with no loss of affinity. Moreover, N/OFQ_ATTO594_ displayed no activity at MOP, DOP or KOP and the ligand retained full biological activity in experiments to measure inhibition of cAMP formation and ERK1/2 phosphorylation. N/OFQ_ATTO594_ was used to visualise receptor location in recombinant and native (immune cells) NOP expression systems [15]. A further difference between the binding of Derm_ATTO488_ and N/OFQ_ATTO594_ relates to NSB. In the present study we report NSB for the Dermorphin ligand between 16 and 19%; for N/OFQ ligand the NSB was effectively undetectable in the conditions used.

There is extensive evidence for opioid dimers in both in vitro recombinant expression systems and in vivo [23–25]. Use of both N/OFQ_ATTO594_ (red) and DermA_TTO488_ (green) in double expression systems where there is a possibility to use FRET to visualize NOP-MOP dimers will an interesting further use of these new ligands. Studies with these new fluorescent ligands as agonists offer the possibility to examine receptor trafficking following activation of the receptor.

There have been other studies using opioid peptides conjugated to other fluorescent ‘probes’. Gaudriault et al (1997) synthesized BODIPY derivatives of Dermorphin and the DOP peptide agonist Deltorphin-I. These probes were used in COS cells transfected with rat MOP and DOP receptors. The authors used these ligands in internalisation studies of live cells and showed that both were capable of visualising receptors and that receptor (probe) internalisation was time and temperature dependent [26]. A range of fluorescent peptide agonists including Dermorphin-Bodipy Texas Red and Dermorphin_Alexa488_ were synthesized by Arttamangkul et al. In radioligand binding studies using CHO cells expressing rat MOP and DOP the authors reported good MOP selectivity; no data are presented for KOP or NOP. Functional activity was confirmed electrophysiologically in rat locus ceruleus. Importantly at low temperature (4–8°C) binding measured by confocal microscopy in CHO cells was located to the membrane that internalized on warming (32–35°C). Interestingly, Alexa488 conjugated to the DOP antagonist TIPP bound but failed to internalize [27]. Further use of these Dermorphin-Bodipy Texas Red and Dermorphin_Alexa488_ probes in primary cultures of mouse locus ceruleus showed that desensitization is not dependent on receptor internalization [28].

There are a number of drawbacks to Derm_ATTO488_. First, Dermorphin is an agonist and as such it will produce receptor activation; we have clearly shown that in this paper. In imaging experiments where cell surface receptor expression is critical activation will lead to Arrestin recruitment, we have shown this previously [14] and ultimately internalization. As such confocal experiments need to be performed at 4°C to limit this process. Whilst a clear disadvantage for studies examining internalisation, protocols to warm cells should enable the internalisation process to be tracked. The second major drawback (no issue in single recombinant expression systems) relates to selectivity of only ~10 fold over DOP noted above. In native tissues aimed at exploring MOP receptor expression, DOP receptors are also often co-expressed so careful use of label concentration is needed to allow discrimination or pre-blocking with highly selective DOP antagonists such as naltrindole.

In summary Derm_ATTO488_ represents a useful addition to the ligand toolkit to study opioid receptor expression, function and turnover.

## Supporting information

S1 File (DOCX)
