# Supplementary material for: Evaluation of [Cys(ATTO 488)8]Dermorphin-NH2 as a novel tool for the study of μ-opioid peptide receptors
Source: PLoS One. 2021 Apr 23;16(4):e0250011. doi: 10.1371/journal.pone.0250011 (PMC8064508; doi:10.1371/journal.pone.0250011)
Supplement: S1 File — (DOCX) [file pone.0250011.s001.docx]

# **S1 File.**

**SUPPLEMENT – CHEMISTRY**

Analytical HPLC analyses was performed on a Beckman 116 liquid chromatograph equipped with a Beckman 166 diode array detector. The purity of Derm_ATTO488_ was monitored at 220 nm and assessed with a Symmetry® C18 column (4.6 x 75 mm, 3.5μm particle size) at a flow rate of 0.5 mL/min using a linear gradient from 100% of A (water + 0.1% trifluoacetic acid) to 100% of B (acetonitrile + 0.1% trifluoacetic acid) over a period of 25 minutes. The molecular weight of the conjugated peptide was confirmed by ESI Micromass ZQ, Waters. Analytical HPLC chromatogram and ESI mass spectra of Derm_ATTO488_ are shown in Fig S1 and Fig S2.

**S1 Fig. Analytical HPLC profile of Derm_ATTO488_**.


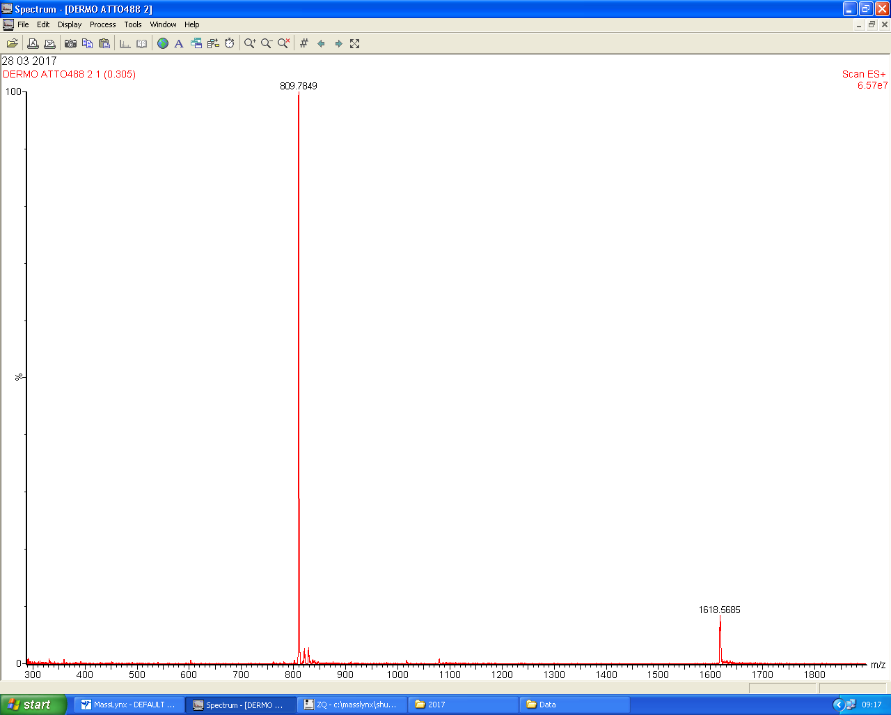


**S2 Fig. MS spectra of Derm_ATTO488_. Peaks at 1618.5685 Da and 809.7849 Da correspond to the [M+H]^+^ and [M+2H]^2+^ molecular ions respectively of the desired product**.


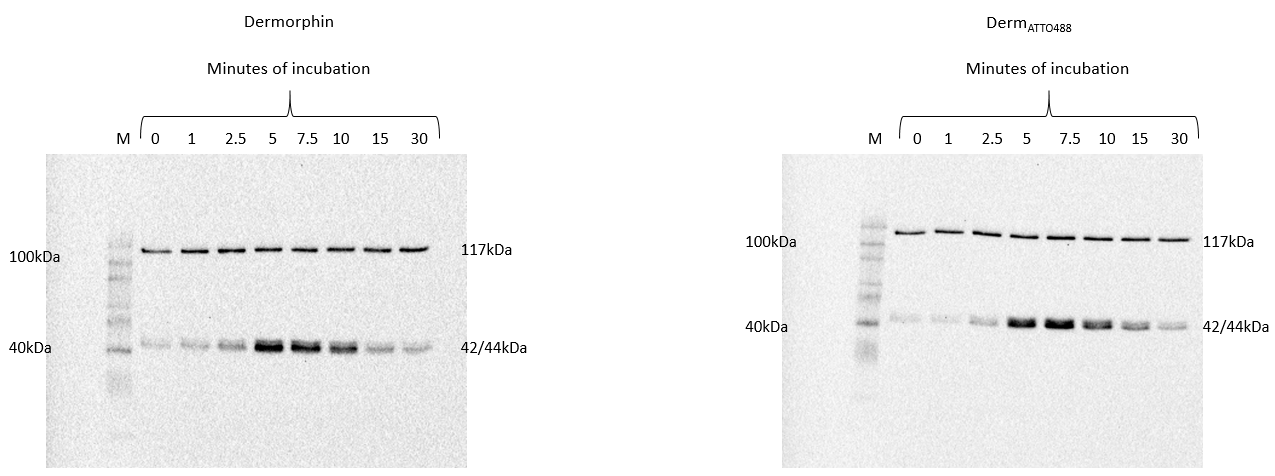


**S3 Fig. Activation of ERK1/2.** Uncropped and unadjusted images of the representative blots for stimulation of phosphorylated ERK1/2 in HEK_MOP_ by Dermorphin and Derm_ATTO488_. The ~28kDa band corresponds to phosphorylated ERK1/2; the 117kDa band represents the vinculin protein.


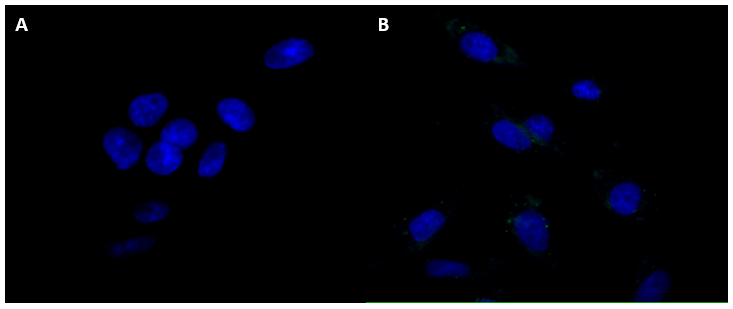


**S4 Fig. WT CHO treated with Derm_ATTO488_ and NSB at MOP with Naloxone**. (A) In untrasfected wild-type CHO cells 1µM Derm_ATTO488_ bound at very low levels, comparable to that defined by (B) Naloxone (15 µM) in CHO_hMOP_.


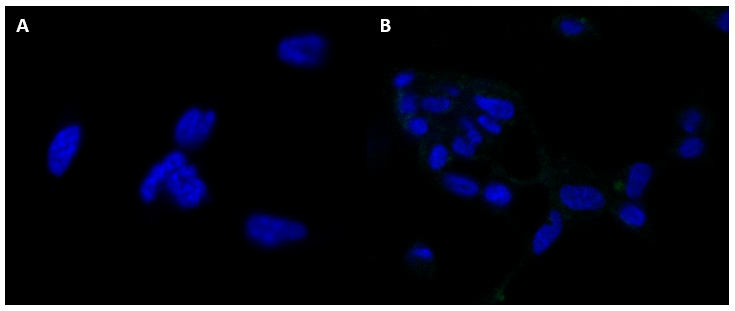


**S5 Fig. WT HEK cells treated with Derm_ATTO488_ and NSB at MOP with Naloxone**. (A) Low levels of binding were observed after treating wild-type HEK_hMOP_ cells with DermATTO488 (1µM). (B) Pre-incubation of HEK_hMOP_ with Naloxone (15µM) defined non-specific binding.
